# Characterisation of ethylene pathway components in non-climacteric capsicum

**DOI:** 10.1186/1471-2229-13-191

**Published:** 2013-11-28

**Authors:** Wan M Aizat, Jason A Able, James CR Stangoulis, Amanda J Able

**Affiliations:** 1 School of Agriculture, Food and Wine, Waite Research Institute, The University of Adelaide, Glen Osmond SA 5064, Australia; 2 School of Biological Science, Flinders University, Bedford Park SA 5042, Australia

**Keywords:** Capsicum, Ethylene pathway, Non-climacteric fruit, Ripening

## Abstract

**Background:**

Climacteric fruit exhibit high ethylene and respiration levels during ripening but these levels are limited in non-climacteric fruit. Even though capsicum is in the same family as the well-characterised climacteric tomato (Solanaceae), it is non-climacteric and does not ripen normally in response to ethylene or if harvested when mature green. However, ripening progresses normally in capsicum fruit when they are harvested during or after what is called the ‘Breaker stage’. Whether ethylene, and components of the ethylene pathway such as 1-aminocyclopropane 1-carboxylate (ACC) oxidase (ACO), ACC synthase (ACS) and the ethylene receptor (ETR), contribute to non-climacteric ripening in capsicum has not been studied in detail. To elucidate the behaviour of ethylene pathway components in capsicum during ripening, further analysis is therefore needed. The effects of ethylene or inhibitors of ethylene perception, such as 1-methylcyclopropene, on capsicum fruit ripening and the ethylene pathway components may also shed some light on the role of ethylene in non-climacteric ripening.

**Results:**

The expression of several isoforms of *ACO*, *ACS* and *ETR* were limited during capsicum ripening except one *ACO* isoform (*CaACO4*). ACS activity and ACC content were also low in capsicum despite the increase in ACO activity during the onset of ripening. Ethylene did not stimulate capsicum ripening but 1-methylcyclopropene treatment delayed the ripening of Breaker-harvested fruit. Some of the *ACO*, *ACS* and *ETR* isoforms were also differentially expressed upon treatment with ethylene or 1-methylcyclopropene.

**Conclusions:**

ACS activity may be the rate limiting step in the ethylene pathway of capsicum which restricts ACC content. The differential expression of several ethylene pathway components during ripening and upon ethylene or 1-methylclopropene treatment suggests that the ethylene pathway may be regulated differently in non-climacteric capsicum compared to the climacteric tomato. Ethylene independent pathways may also exist in non-climacteric ripening as evidenced by the up-regulation of *CaACO4* during ripening onset despite being negatively regulated by ethylene exposure. However, some level of ethylene perception may still be needed to induce ripening especially during the Breaker stage. A model of capsicum ripening is also presented to illustrate the probable role of ethylene in this non-climacteric fruit.

## Background

Fruit can be divided into two different ripening behaviours, climacteric and non-climacteric types. Climacteric fruit such as banana and tomato generally exhibit ethylene and respiration surges during ripening but non-climacteric fruit such as grapes and capsicum do not [1-3]. The ethylene hormone also regulates the ripening rate of climacteric fruit but its function during non-climacteric ripening is still inadequately understood [4,5]. Ethylene is produced from the 1-aminocyclopropane 1-carboxylate (ACC) precursor by the action of the ACC oxidase (ACO) enzyme [1]. ACC is synthesised by ACC synthase (ACS) from S-adenosyl methionine (SAM) which originates from the amino acid methionine [1]. Differences between the capability of climacteric and non-climacteric fruit to produce ethylene probably lie with the presence of two systems of ethylene production exclusively in climacteric fruit [4,6].

During fruit development, ethylene is produced at a basal level in climacteric and non-climacteric fruit alike, a process known as System 1 [4,6]. When climacteric fruit reach maturity, another process called System 2 is initiated to produce a burst in ethylene production to promote ripening while non-climacteric fruit are thought to remain in System 1 [4,6,7]. The regulation of these two systems has been associated with the differential expression of *ACO* and *ACS* isoforms, especially when first characterised during tomato ripening [8,9]. There are at least six *ACO* isoforms in tomato and nine known *ACS* isoforms but only some of them are expressed during ripening to regulate the two systems [2,10]. For example, *LeACS1A* and *LeACS6* were expressed during System 1 ethylene production and subsequently, *LeACS2* and *LeACS4* as well as *LeACO1* were highly induced during System 2 ethylene production. Furthermore, System 1 is also known to be an auto-inhibitory system whereas System 2 is an auto-stimulatory system [1,4]. In climacteric tomato, System 1-associated isoforms (such as *LeACS1A*) are negatively regulated by high ethylene whereas System 2-associated isoforms (such as *LeACO1* and *LeACS2*) are positively regulated [6,8]. Given that these *ACO* and *ACS* isoforms were regulated by the presence of ethylene, its perception also appears integral to climacteric ripening. Indeed, *ethylene receptors* (*ETR*s) have been shown to be differentially regulated during ripening and upon ethylene treatment [11]. The six tomato ETR isoforms can also be classified into two subfamilies, subfamily I (LeETR1, LeETR2, LeETR3) and subfamily II (LeETR4, LeETR5, LeETR6), with possible differences between these groups explained by their affinity towards the downstream protein, Constitutive Triple Response 1 (CTR1) [12,13]. However, the regulation of these isoforms and the two ethylene production systems during non-climacteric ripening is still inadequately documented and hence further research is needed.

Given that capsicum belongs to the Solanaceae family and shares genetic similarity with tomato, the characterisation of the same ethylene pathway in non-climacteric capsicum will enhance our understanding of differences in ethylene production in the two ripening types. Earlier microarray studies have reported that transcripts associated with ethylene signalling were up-regulated in both capsicum and tomato ripening [14,15]. Our recent proteomic analysis also revealed that during capsicum ripening, an ACO protein isoform 4 (CaACO4) was increased (which corresponded to the ACO activity and mRNA expression), suggesting a conserved ethylene pathway may be involved in the ripening of this non-climacteric fruit [16]. However, further components of this pathway such as other *ACO* isoforms, *ACS* and *ETR* isoforms and their regulation in capsicum are still not well described. Additionally, capsicum exhibits a unique ripening behaviour when harvested off the plant; only ripening properly when harvested at Breaker or later but not when harvested during the Green stage [17]. This suggests ripening regulators may be present exclusively during Breaker stage onwards to induce ripening in non-climacteric capsicum, possibly in an ethylene independent pathway (as ripening can proceed without high levels of ethylene production). Therefore, further post-harvest studies employing ethylene or 1-methylcyclopropene (1-MCP) treatment of both Green and Breaker stages are necessary to characterise the ethylene pathway and/or the possible involvement of ethylene independent pathways in the non-climacteric ripening of capsicum.

In this study we have investigated the expression of *ACO*, *ACS* and *ETR* isoforms during capsicum (*Capsicum annuum* cv. Aries) ripening using quantitative real-time PCR (qPCR) at six different ripening stages (Green, G; Breaker, B; Breaker Red 1, BR1; Breaker Red 2, BR2; Light Red, LR; Deep Red, DR). ACS activity and ACC content during the ripening stages were also examined to contrast their levels with climacteric fruit. Furthermore, capsicum was treated with ethylene or 1-MCP at two different stages of ripening (G and B) and their effect on ripening, ACO and ACS activity, and ACC content was analysed during post-harvest storage. The expression of *CaACO*, *CaACS* and *CaETR* isoforms directly after treatment was also studied.

## Results

### CaACO, CaACS and CaETR isoforms were differentially expressed during capsicum ripening

**Figure 1:**
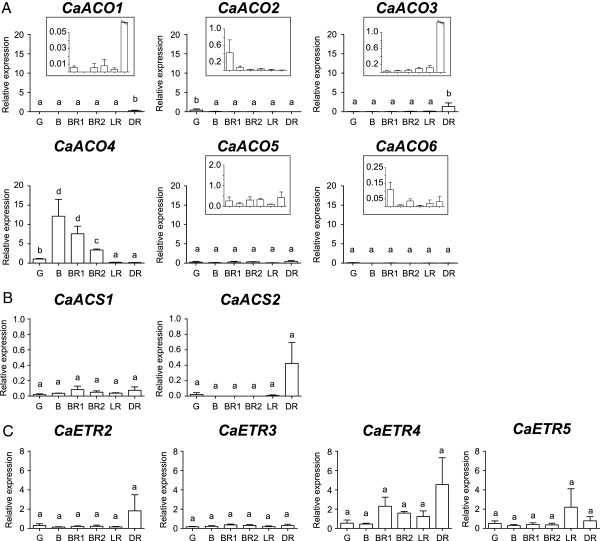
**Gene expression of *CaACO *(A), *CaACS *(B) and *CaETR *(C) isoforms during capsicum ripening as determined by qPCR.** G, Green; B, Breaker; BR1, Breaker Red 1; BR2, Breaker Red 2; LR, Light Red; DR, Deep Red. Bars represent the mean ± SE of n = 3 biological replicates. The same letter indicates no difference between means as determined using the Least Significant Difference (*P* < 0.05). Gene expression was normalised relative to *CaGAPdH* expression according to the Methods. Note that the relative expression axis was set at a similar value for *CaACO*, *CaACS* and *CaETR*, respectively. For *CaACO1*, *CaACO2*, *CaACO3*, *CaACO5* and *CaACO6*, inset figures are shown with respect to their relative expression values corresponding to the ripening stages (G, B, BR1, BR2, LR, DR) and the double slash on both DR stage bars of *CaACO1* and *CaACO3* indicate values higher than the maximum **(A)**.

Throughout capsicum ripening, the transcript expression of most *ACO* isoforms was limited except *CaACO4* (Figure 1A). *CaACO4* relative expression (normalised by *CaGAPdH*) was significantly greater during ripening onset (approximately seven to 12 times at B and BR1 compared to G) with minimal expression during the full red stages (LR and DR). Even though *CaACO1* and *CaACO3* transcripts were significantly increased at the DR stage and *CaACO2* was increased at the G stage, their relative expression levels throughout capsicum ripening stages were still very low compared to *CaACO4*. The relative transcript expression of *CaACO5* and *CaACO6* was also extremely low but constant during ripening.

Both *CaACS1* and *CaACS2* were not highly expressed during ripening relative to *CaGAPdH* (Figure 1B). The gene expression of both isoforms was also not significantly different during ripening but *CaACS1* was expressed more constantly throughout the six stages compared to *CaACS2*.

No significant change in the gene expression of any *ETR* isoforms was measured during ripening (Figure 1C). Comparing their levels, *CaETR4* was the main isoform expressed during capsicum ripening. *CaETR4* appeared to slightly increase from G (~0.5 relative expression) to the BR1 (~2.3 relative expression) and DR stages (~4.5 relative expression), but this was not statistically significant. In comparison, the expression of *CaETR2*, *CaETR3* and *CaETR5* was consistently low (mostly less than 2.0 mean relative expression) in all stages of ripening.

### ACS activity and ACC content were limited during capsicum ripening

**Figure 2:**
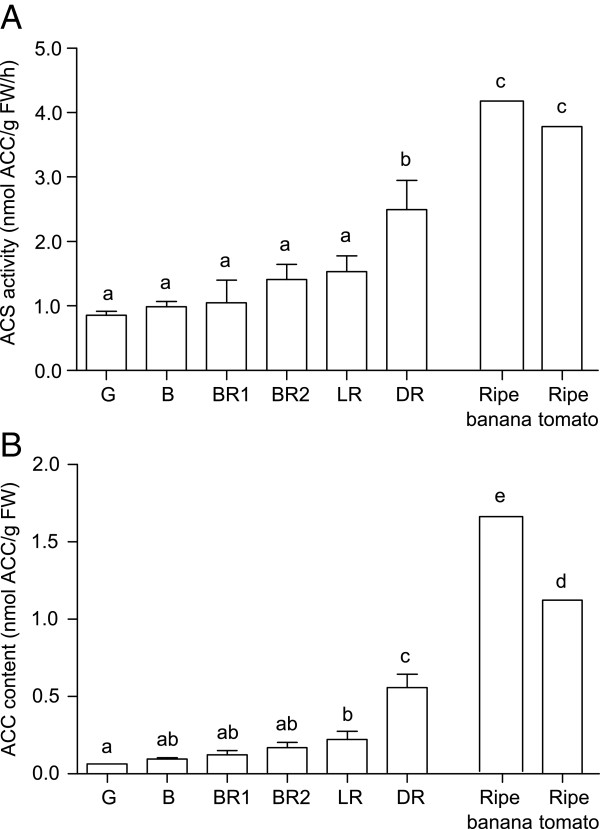
**ACS activity (A) and ACC content (B) during the six stages of capsicum ripening.** G, Green; B, Breaker, BR1, Breaker Red 1; BR2, Breaker Red 2; LR, Light Red; DR, Deep Red. ACS activity is expressed as nmol ACC/g fresh weight (FW)/ h and the ACC content is expressed as nmol ACC/g FW. Bars represent means (±SE) for n = 3 biological replicates for all capsicum ripening stages and n = 1 biological replicate for ripe banana and tomato (as positive controls where their levels have been similarly reported in other studies [18,19]). The same letter indicates no difference between means as determined using the Least Significant Difference (*P* < 0.05).

The mean level of ACS activity (Figure 2A) was not significantly different among any of the stages between G and LR but increased significantly at the DR stage by approximately two-fold. ACS activity in capsicum was approximately two to four times lower than the two positive climacteric controls (ripe banana and tomato). The level of ACC content (Figure 2B) only increased significantly from the G stage to the LR and DR stages. The amount of ACC in ripe banana and tomato was approximately seven times higher than the average ACC level throughout capsicum ripening. Furthermore, the levels of ACS activity and ACC content reported here for banana and tomato as well as capsicum, correspond to other previous reports [18-20].

### The effects of ethylene or 1-MCP treatment

**Figure 3:**
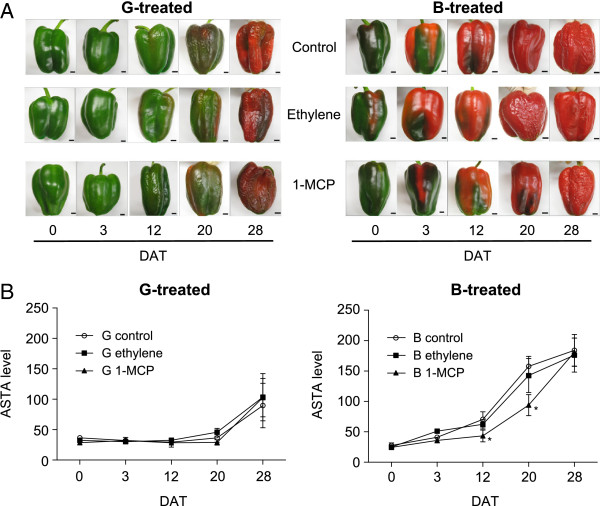
**Treatment of Green (G, left) and Breaker (B, right) harvested capsicum with 100 μL/L ethylene or 500 nL/L 1-MCP (for 24 h).** Untreated control fruit were also prepared for both stages. **A)** Ripening of G and B treated fruit during storage after treatment. Representative images are shown. The scale at the bottom right of the pictures is 1 cm. **B)** The extractable colour (ASTA units) from each treatment and DAT (days after treatment). Asterisks adjacent to symbols indicate means were significantly different compared to the respective control at each DAT as determined by a Duncan’s Multiple Range Test (*P* < 0.05) (Additional file 4: Table S2 for details). Means are n = 3 biological replicates (±SE).

Untreated B-harvested fruit ripened normally and developed to DR after 28 days in storage but untreated G-harvested fruit ripened incompletely (Figure 3A). Regardless of sampling time during storage, ethylene or 1-MCP treatment did not significantly affect the ripening behaviour (Figure 3A, left) and colour development (Figure 3B, left) of G-harvested capsicum. The extractable colour of G-harvested capsicum in all treatments and the control was slightly increased at 28 days after treatment (DAT) compared to earlier sampling times (Figure 3B, left) but was still lower than the fully red coloured capsicum of untreated B-harvested control fruit (approximately 140 ASTA units at 20 DAT, Figure 3B, right). This amount of extractable colour was also achieved by others [17], confirming our present result. In contrast, the ripening behaviour of 1-MCP treated B-harvested fruit was shown to be delayed compared to those treated with ethylene or the control especially from 3 to 20 DAT (obvious green/black tissues, Figure 3A, right). The extractable colour of both control and ethylene treated fruit reached 140 ASTA units at 20 DAT whereas the 1-MCP treated fruit did not do so until 28 DAT (Figure 3B, right). Regardless of sampling time, the ethylene treatment of B fruit did not significantly affect extractable colour but 1-MCP treatment significantly reduced it at 12 and 20 DAT (Figure 3B right). In terms of percentage weight loss, there were no significant differences regardless of treatment or when fruit was harvested but there was an obvious increase in the percentage weight loss throughout sampling (up to 30% weight loss at the 28 DAT for both G and B treated fruit) indicating that the fruit were dehydrated over time (data not shown).

**Figure 4:**
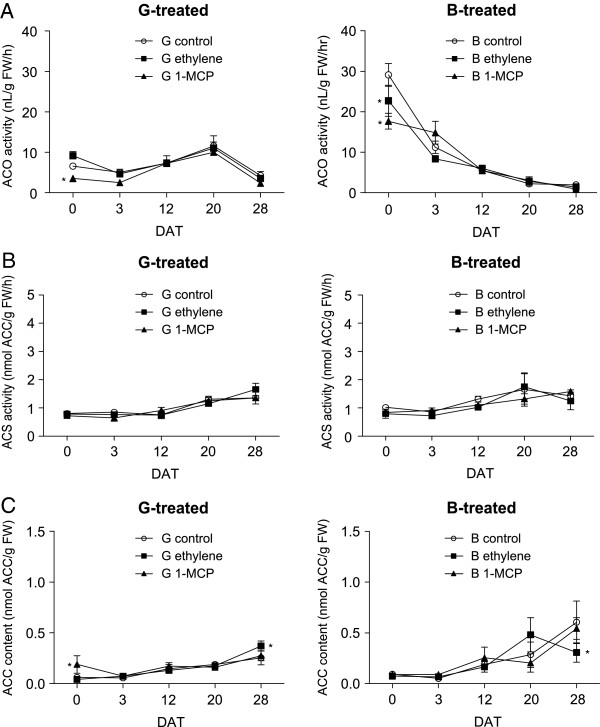
**ACO activity (A), ACS activity (B) and ACC content (C) of capsicum treated with 100 μL/L ethylene or 500 nL/L 1-MCP (for 24 h) at two ripening stages, Green-harvested and treated (left) and Breaker-harvested and treated (right).** Untreated control fruit were also analysed for both stages. Asterisks adjacent to symbols indicate means were significantly different compared to the respective control at each DAT (days after treatment) as determined by Duncan’s Multiple Range Test (*P* < 0.05) (Additional file 4: Table S2 for details). Means are n = 3 biological replicates (±SE).

In our previous report, the ACO activity of capsicum increased significantly at B-BR1 stages compared to the other ripening stages [16]. Indeed, the ACO activity reported here was also significantly higher in untreated B-harvested control fruit than untreated G-harvested control fruit at 0 DAT (Figure 4). Furthermore, at 0 DAT, the ACO activity of G fruit treated with ethylene was not significantly greater than the control while 1-MCP treated fruit had significantly lower ACO activity than the control (Figure 4A). On the other hand, the ACO activity of B-harvested fruit treated with ethylene or 1-MCP was significantly lower than the activity of the untreated B-harvested control fruit. Throughout storage, regardless of treatment, ACO activity peaked slightly at 20 DAT in G-harvested fruit while ACO activity in B-harvested fruit generally exhibited a downward trend (Figure 4).

The ACS activity of both G-harvested and B-harvested fruit (Figure 4B left and right, respectively) was not significantly different, regardless of sampling time and treatment, and significantly lower than the banana positive control (similar level as in Figure 2A, data not shown).

Regardless of sampling time during storage, the ACC content of both G-harvested and B-harvested fruit (Figure 4C left and right, respectively) was consistently lower than the banana positive control (similar level as in Figure 2B, data not shown). At 0 DAT, G-harvested fruit treated with 1-MCP had significantly greater ACC content compared to the control and ethylene-treated fruit. Furthermore, at 28 DAT, there was more ACC in G-harvested ethylene-treated fruit compared to the untreated control and *vice versa* for B-harvested fruit. However, these ACC levels (control and ethylene-treated fruit at 28 DAT) of both G and B harvested capsicum were not significantly different compared to 1-MCP-treated fruit. No other significant changes were observed between the control and treatments at any other DAT.

### Differential expression of CaACO, CaACS and CaETR isoforms directly after treatment (0 DAT) with ethylene or 1-MCP

**Figure 5:**
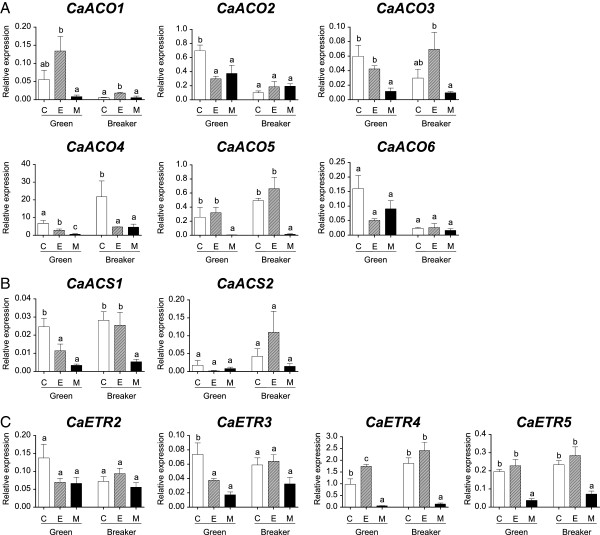
**The qPCR analysis of *CaACO *(A), *CaACS *(B) and *CaETR *(C) isoforms of Green and Breaker treated capsicum at 0 DAT. C**, Control (open bars); E, 100 μL/L ethylene treatment for 24 h (striped bars); M, 500 nL/L 1-MCP treatment for 24 h (closed bars). Significantly different levels were determined using Least Significant Difference (*P* < 0.05) of 1-way ANOVA at each stage (Green and Breaker, respectively) and are indicated by different letters on bars (±SE of n = 3 biological replicates). Gene expression was normalised relative to *CaGAPdH* expression according to the Methods. Note that the relative expression axis was set according to respective isoforms.

The six *CaACO* isoforms of capsicum exhibited differential expression upon ethylene or 1-MCP treatment (Figure 5A). When comparing control and ethylene-treated samples of G-harvested fruit, *CaACO1* was not statistically significant. However, the relative expression of *CaACO2* and *CaACO4* was significantly lower (approximately two-fold) in the ethylene-treated samples compared to the respective control. Moreover, the relative expression of other isoforms such as *CaACO3*, *CaACO5* and *CaACO6* was not significantly different between ethylene-treated and control G fruit samples. Conversely, G-harvested fruit treated with 1-MCP had significantly lower *CaACO2, CaACO3, CaACO4* and *CaACO5* expression compared to the control. Among these isoforms, *CaACO5* was affected the most by 1-MCP (approximately 52-fold lower expression than the control) while others had only approximately two to 11-fold differences. The 1-MCP treatment however did not significantly change *CaACO1* or *CaACO6* expression of G-harvested capsicum. For B-harvested capsicum, the relative expression of ethylene-treated samples was significantly higher for *CaACO1* but significantly lower for *CaACO4* compared to the respective control*.* Ethylene had no effect on the expression of *CaACO2*, *CaACO3, CaACO5* and *CaACO6* while 1-MCP had no effect on *CaACO1*, *CaACO2*, *CaACO3,* and *CaACO6* in B-harvested fruit. In addition, 1-MCP treatment of B fruit caused *CaACO4* to be significantly lower than the control by three to five-fold and more so for *CaACO5* (approximately 33-fold less in treated samples). When compared across the different *ACO* isoforms within a particular treatment/control, *CaACO4* had the highest relative expression.

For the isolated *CaACS* isoforms (Figure 5B), both isoforms also showed differential expression upon treatment. *CaACS1* expression was significantly reduced in the ethylene-treated G fruit (approximately two-fold less relative expression compared to the untreated G fruit control) and in 1-MCP-treated samples of both G and B harvested fruit (approximately seven-fold less relative expression compared to the respective control). No significant relative expression differences were observed for *CaACS2* between treated samples and the control, where fruit was harvested at either ripening stage.

For the four *CaETR* isoforms (Figure 5C), different expression patterns were observed. Firstly and regardless of the ripening stage that fruit were harvested and the treatment, no significant changes were measured for *CaETR2*. However, ethylene or 1-MCP treatment of G-harvested fruit did result in significantly lower *CaETR3* relative expression (two to four-fold), while no significant differences were observed in B-harvested fruit for the two treatments. Moreover, the relative expression of *CaETR4* upon ethylene treatment at the G stage was significantly higher by approximately two-fold compared to the control while 1-MCP treatment caused *CaETR4* relative expression to be lower by approximately 17-fold when compared to the G-harvested control. However, no significant difference in *CaETR4* expression between ethylene-treated and control fruit was observed when they had been treated at the B stage. Nonetheless in B-harvested fruit, *CaETR4* relative expression was significantly lower by approximately 14-fold when 1-MCP-treated fruit were compared with the control. For *CaETR5* expression, no significant difference was observed between the control and ethylene-treated samples in both G and B fruit. However, 1-MCP treatment caused the *CaACO5* transcript level to significantly drop by approximately three to five-fold in both ripening stages compared to the control. In summary and when comparing different *ETR* isoforms within a particular treatment/control, *CaETR4* still had the highest relative expression (except G-harvested samples treated with 1-MCP which were similar for *CaETR4* and *CaETR2*).

## Discussion

The molecular mechanisms of capsicum ripening are inadequately understood, particularly for non-climacteric behaviour. Due to having genetic similarities with the model fruit tomato, capsicum may become a useful resource to elucidate the molecular regulation of non-climacteric ripening, especially with regards to the ethylene pathway. In this study we have demonstrated that ACS activity and ACC content were limited in capsicum while several *ACO*, *ACS* and *ETR* isoforms were differentially regulated upon ripening and ethylene treatment. Furthermore, 1-MCP treatment during the onset of ripening (B stage) significantly delayed ripening and reduced the expression of several isoforms, indicating that ethylene perception may be required, to some extent, for non-climacteric fruit ripening to occur.

### Rate limiting ACS activity during capsicum ripening affects the level of its product, ACC

The presence of ACS protein appears to be the rate limiting step in the ethylene pathway of capsicum. Even though ACO activity was greater in B fruit than other ripening stages (Figure 4A, [16]) and the level in capsicum seems to be comparable with the climacteric tomato during ripening [21,22]; capsicum ACS activity was approximately two to four-fold lower than that for the climacteric fruit used as positive controls (tomato and banana, Figure 2A). In addition, the pattern of *CaACS1* expression corresponded well with the basal level of ACS activity during G to LR, and the increase of ACS activity during DR (Figure 2A) may be due to both *CaACS1* and *CaACS2* expression at the same time (Figure 1B). An earlier study on *CaACS1* also showed that its expression was minimal but constant throughout capsicum ripening stages [23], thus corroborating our current findings. Furthermore, ACS activity in climacteric fruit has been shown to be increased during ripening onset [24,25] but its level in capsicum remained constant for most of the ripening stages (Figure 2A), suggesting that ACS was the rate limiting step in this non-climacteric fruit.

The level of ACC, the product formed from SAM by the action of ACS, was also very low in capsicum such that there was on average, seven-fold less ACC during capsicum ripening compared to the climacteric tomato and banana (Figure 2B). The level of ACC content in capsicum has previously been shown to be limited [26] in a similar manner as other non-climacteric fruit including strawberry [27] and grapes [28]. This is further corroborated by the significant increase in ACC level observed during the LR and DR stages (Figure 2B), probably in response to the limited ACO activity [16] preventing its conversion to ethylene. Furthermore, reducing the amount of ACO activity significantly by means of 1-MCP application during the G stage (at 0 DAT) also increased ACC content (Figure 4A and C, left). This confirms that not only ACC is limited in capsicum but that it might also be continually required for the basal level of ethylene production. However, no increase in ACC content was observed in B fruit directly after 1-MCP treatment despite the reduction in ACO activity, probably because the ACO activity level was still high in B-treated fruit compared to G-treated fruit (Figure 4). This suggests that the low levels of ethylene produced (approximately 0.1 nL/g fresh weight (FW)/h for our capsicum compared to 1.5-3 nL/g FW/h ethylene production for the banana and tomato used as controls in this study; data not shown) was probably due to the lack of precursors for ethylene production caused by the restricted ACS activity.

### Differential expression of systems 1 and 2-associated isoforms in non-climacteric capsicum ripening

The ripening of climacteric tomato is generally accompanied by a burst in ethylene production which is caused by System 2. The transition from the negative feedback mechanism of System 1 during fruit development to the positive feedback mechanism of System 2 during the ripening of mature fruit can be regulated by the differential expression of specific isoforms in the ethylene pathway [1]. For example, *LeACS1A* and *LeACS6* are considered to be System 1 components as they are expressed in immature fruit and are negatively regulated by ethylene treatment [8,9]. Once fruit reach maturity, *LeACS2* and *LeACS4* are highly expressed during ripening and upon ethylene treatment, suggesting that they are System 2-associated isoforms [8,22]. The climacteric increase in ethylene production during System 2 was also supported by the up-regulation of *ACO* isoforms particularly *LeACO1*, and to some extent *LeACO4*[9,29,30]. The application of ethylene to the mature G stage not only significantly increased these *ACO* isoforms [22,31] but also induced the expression of *ETR* isoforms, primarily *LeETR3*, *LeETR4* and *LeETR6*[11]. The regulation of the two ethylene production systems in non-climacteric fruit is however still not fully described but System 1 is generally considered to be operating throughout ripening [4,7].

Indeed, the expression and regulation of the ethylene pathway components in non-climacteric capsicum were somewhat different compared to the climacteric tomato. For instance, *CaACS2* was not highly expressed during ripening (Figure 1B) and upon ethylene treatment (Figure 5B), while *CaETR4* and *CaACO1* did respond to ethylene but only to some extent in either G or B-harvested fruit, respectively (Figure 5A and C). *CaACO1* expression was also limited throughout capsicum ripening (Figure 1A) which confirmed earlier reports [14,32]. The failure of *CaACO1* and *CaACS2* to be highly stimulated upon ripening and ethylene exposure as in climacteric tomato may suggest the absence of System 2, thus the burst in ethylene production associated with this system could not be induced in this non-climacteric fruit. Furthermore, *CaACO4* could be considered the major isoform expressed during capsicum ripening onset (B-BR stages) as no other *ACO* isoform exhibited similar levels of up-regulation (Figure 1A). This is in good agreement with our previous report on *CaACO4* expression using semi-quantitative RT-PCR and the overall ACO activity level during capsicum ripening [16]. Interestingly, *CaACO4* was negatively regulated by ethylene (Figure 5A) particularly in B-harvested fruit suggesting its negative regulation may result in the lower overall ACO activity observed after ethylene treatment when compared to the untreated control fruit (Figure 4A, right). Other isoforms such as *CaACO2*, *CaACS1* and *CaETR3* were also down-regulated upon ethylene treatment particularly at the G stage (Figure 5), which implies that System 1 may be predominantly operating in this non-climacteric fruit ripening rather than System 2.

The down-regulation of several transcripts upon ethylene treatment also suggests that other ripening regulators that might control their expression during capsicum ripening. These ripening regulators might also be present exclusively during the B stage as G-harvested fruit did not ripen properly and the ACO activity of G-harvested capsicum cannot be induced to the level of B capsicum even with ethylene treatment (Figure 4A). *CaACO4*, which may be considered to be a System 1-associated isoform, was up-regulated during ripening onset (Figure 1A) which suggests that its up-regulation might be closely associated with these ripening regulators in an ethylene-independent manner. Two pathways, ethylene dependent- and independent- pathways, have been suggested to operate in climacteric fruit but only the latter pathway may be conserved in non-climacteric fruit to induce ripening [6,33]. The main regulators of the ethylene-independent pathways especially in non-climacteric fruit are still unknown but the *Le*MADS-RIN transcription factor (RIN) has been proposed to be one of the regulators [1,34]. RIN has been shown to control ripening even prior to the climacteric ethylene production in tomato, suggesting that this transcription factor may sit upstream of the ethylene pathway [33,35]. Indeed, RIN has been shown to regulate, directly or indirectly, the expression of several ethylene pathway components including *LeACS2* and *LeACO1*[1,36-38]. Interestingly, the expression of the two isoform homologues in capsicum, *CaACS2* and *CaACO1*, were limited (Figure 1). Furthermore, *LeACO4* may be considered to be a System 2-associated isoform [22] in contrast to the System 1-associated *CaACO4* (Figure 1A). This difference might be attributable to genetic rearrangement that heavily occurred in capsicum when compared with tomato [39], such that upstream promoter regulation is no longer similar between these (and possibly other) Solanaceae members. Further investigation is therefore needed to compare the isoform promoter regions of both fruit as well as the possible involvement of RIN or other ripening regulators in the ethylene-independent pathway(s) of capsicum ripening.

### Ethylene perception may be partially required in capsicum ripening especially during ripening onset

Although the ethylene independent pathway(s) may exist in non-climacteric ripening, our results also highlighted that ethylene perception may still be needed for capsicum ripening, to some extent. 1-MCP treatment, which blocks ethylene perception, delayed the ripening rate of B capsicum by approximately seven days (Figure 3A and B). The application of 1-MCP on other non-climacteric fruit such as grapes and strawberry also has been shown to slow some ripening aspects [28,40,41]. Furthermore, subfamily II receptors, *CaETR4* and *CaETR5* were significantly less in 1-MCP-treated samples compared to the control (Figure 5C), which may consequently influence the down-regulation of other downstream ethylene pathway components such as *CaACS1*, *CaACO4* and *CaACO5* (Figure 5A, B). Subfamily II *CaETR4* may also be considered to be the major *ETR* isoform expressed in capsicum due to its higher overall relative expression during ripening (Figure 1C). Interestingly, in strawberry, *FaETR2* which is closely related to *LeETR4* was also the major isoform expressed [12]. Since both CaETR4 and FaETR2 belong to subfamily II ETRs which may have weaker binding with the CTR1 protein [13], the basal level of ethylene in the non-climacteric fruit may be sufficient to induce considerable changes to downstream ethylene responses and the inhibition of the ethylene perception may also severely impact ripening.

Ethylene binding has been associated with controlling the rate of ethylene receptor turnover in tomato [11], and in the case of non-climacteric fruit, the basal level of ethylene may be needed to sustain a certain receptor level and hence maintain ethylene perception for normal ripening to proceed. This was further corroborated by our findings in that ethylene treatment did not induce any significant changes towards ripening (Figure 4A, B), and the expression of all four measured *ETR* isoforms at the B stage in capsicum after ethylene exposure were also constant compared to the control (Figure 5C). This implies that there may be no more receptors available to accept ethylene (as they were possibly saturated) especially during ripening onset and thus there were no effects towards the ethylene perception and response for ripening. This further suggests that only a certain level of ethylene receptors and perception are required for the ripening process, together with the ethylene independent pathway(s) as described earlier. Therefore, a high ethylene level may not be needed and the energy saved can be utilised for other ripening related events such as colour and textural modification. However, any mRNA expression changes (especially for the *ETR*s) need to be confirmed with respective protein expression assays as post-translational regulation has been shown to strictly affect the receptor protein abundance [11]. In addition, given that the *Capsicum chinensis* gene *CcGH3,* which has been shown to influence fruit ripening, is regulated by both ethylene and auxin [42], whether a similar situation is possible for *CaETR* needs to be investigated.

## Conclusions

**Figure 6:**
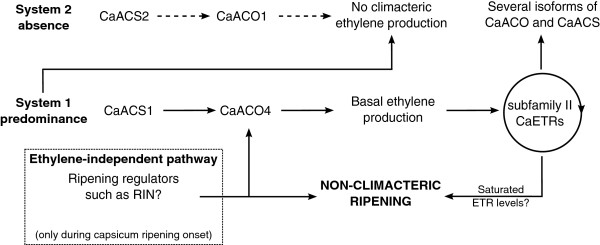
**A proposed model for the ripening of non-climacteric capsicum.** The ethylene pathway generally involves different isoforms of ACO and ACS to produce ethylene before it is perceived by ETR for other downstream responses. In tomato, System 1 ethylene production during development will be followed by System 2 ethylene production to induce climacteric ripening [8]. However, in non-climacteric capsicum ripening, System 2 may be absent based on the limited expression (dotted arrows) of certain System 2-associated isoforms such as *CaACS2* and *CaACO1* during ripening and upon ethylene treatment (compared to their homologue expression in tomato). The expression of *CaACS1* and *CaACO4* during capsicum ripening was associated with System 1 due to their significant reduction upon ethylene treatment at either or both G and B stages. This is in contrast to the *CaACO4* homologue in tomato, *LeACO4,* which is a System 2-associated isoform [22]. Therefore System 1 may be predominantly operating in capsicum to produce the basal ethylene level (while inhibiting System 2 ethylene production). The basal ethylene level may be needed to maintain the rate of ETR turnover, particularly subfamily II CaETR4 and CaETR5, as ethylene perception removal (through 1-MCP treatment) severely affected the *CaETR* expression as well as other possible downstream *CaACO* and *CaACS* isoforms. These subfamily II CaETRs may become saturated, and perhaps together with ethylene independent pathways upon ripening onset, non-climacteric capsicum ripening could be initiated. The ethylene independent pathways may also involve some ripening regulators such as RIN transcription factors and their presence may induce the expression of *CaACO4* upon ripening onset.

Overall, the limited level of ethylene produced in non-climacteric capsicum may be contributed by the rate limiting ACS activity which restricts the ACC content. Furthermore, several isoforms of ethylene-related genes were differentially expressed in capsicum, suggesting alternative regulation and the likelihood that ethylene production in non-climacteric ripening is predominantly by System 1 with System 2 being absent (summarised in Figure 6). Ethylene independent pathway(s) may also be present during capsicum ripening onset but some level of ethylene perception may still be needed for the induction of non-climacteric fruit ripening (Figure 6).

## Methods

### Capsicum ripening tissues and the treatment of G and B harvested fruit with ethylene or 1-MCP

For different ripening stages, tissues from the six capsicum stages (G, B, BR1, BR2, LR and DR) were prepared and described in Aizat et al. [16]. For ethylene and 1-MCP treatments, capsicum plants were grown as per Aizat et al. [16], fruit length was measured every week beginning at 27 days after anthesis (DAA) onwards and harvested on Jan-Feb 2013 (summer season) when fruit reached either G (43 DAA) or B (50 DAA) stage. The average length of the matured stages was approximately 85 mm (data not shown). Harvested fruit at each stage were cleaned using nanopure water, dried at room temperature (RT, 22-23°C) for approximately 1 h, weighed and randomly allocated into nine 10 L plastic containers with a septum (five fruit each container). In each container, there was a heavy-duty towel at the bottom and approximately 100 g Ca(OH)_2_ as a CO_2_ scrubber. Three containers were allocated for each treatment: control (no treatment), ethylene (100 μL/L final concentration, Coregas) or 1-MCP (500 nL/L final concentration, prepared as in Moradinezhad et al. [43]). After a 24 h treatment in the dark at RT, fruit was aired in a laminar flow for 30 min, removed from the containers and placed into aluminium foil trays individually. The fruit were then stored at RT in the dark with potassium permanganate and Ca(OH)_2_ to remove residual ethylene and carbon dioxide respectively [43,44]. At 0 (directly after treatment), 3, 12, 20 and 28 days after treatment (DAT), three fruit for each treatment were weighed, photographed, sampled in liquid N_2_ according to Aizat et al. [16] and stored in −80°C until further analysis.

### cDNA and genomic DNA stocks

For different ripening stages, all cDNA stocks were prepared and described as per Aizat et al. [16]. Genomic DNA was extracted from B fruit for genomic end-point PCR when no products were amplified from the cDNA stocks using a protocol adapted from Karakousis and Langridge [45]. Ground capsicum tissues (250 mg) were homogenised with 0.5 mL DNA extraction buffer pH 8.0 [100 mM Tris–HCl, 100 mM NaCl, 10 mM ethylenediamine tetraacetic acid (EDTA), 1% (w/v) N-Lauroyl Sarcosine (sarkosyl), 1% (w/v) poly(vinylpolypirrolidone) (PVPP)]. Phenol: chloroform: isoamyl alcohol solution (25:24:1 v/v) (0.5 mL) was added, vortexed briefly and mixed on an orbital shaker for 15 min at 4°C. Samples were centrifuged (6000 rpm, 15 min, 4°C) and the upper aqueous solution was added to chloroform (0.5 volume for each 1.0 sample volume). Sample mixtures were then vortexed, centrifuged (13 000 rpm, 10 min, 4°C) and the chloroform extraction was repeated one more time. For every 1.0 mL of final aqueous sample, 90 μL of 3 M sodium acetate and 900 μL isopropanol were then added. The sample was then mixed on an orbital shaker for 10 min at 4°C and centrifuged to pellet (13 000 rpm, 10 min, 4°C). The DNA pellet was washed with 70% ethanol (500 μL) before being air-dried and resuspended in 30 μL sterilised nanopure water. The extracted genomic DNA was quantified using a NanoDrop 1000 (ThermoScientific, Wilmington, DE, USA) as per manufacturer’s instructions.

For different treatments, RNA was extracted from the control and treated capsicum tissues at 0 DAT as per Aizat et al. [16] except that all centrifugation steps were done at 13 000 rpm for 20 min (4°C) and the capsicum materials, as well as buffers used, were scaled down to 1:10. The RNA was quantified, DNAse-treated and synthesised to cDNA according to Aizat et al. [16].

### End-point PCR

All end-point PCR was performed using primers shown in Additional file 1: Table S1, according to Aizat et al. [16] but with some modifications. The template used was cDNA mixture pooled from one biological replicate of all six stages of ripening (cDNA of G, B, BR1, BR2, LR and DR stages), while for all primer pairs the annealing was run at 55°C for 30 s and elongation was run at 72°C for 30 s for all primer pairs. PCR products were transformed into pGEM-T Easy (Promega, Madison, WI, USA) and sequenced from at least four independent colonies as per Aizat et al. [16]. Phylogenetic analysis was performed using Molecular Evolutionary Genetics Analysis (MEGA) program version 5.05 using the conditions stated in Khoo et al. [46].

For genomic end-point PCR the standard PCR procedure above was performed except, with the genomic DNA template, annealing temperatures were either at 55°C, 60°C or 65°C and the elongation step was for 1.5 min to take into account possible introns.

### The identification of CaACO, CaACS and CaETR isoforms in capsicum

Full-length isoform sequences of tomato *ACO* (*LeACO1*, *LeACO2*, *LeACO3*, *LeACO4*, *LeACO5*, *LeACO6*), *ACS* (*LeACS1A*, *LeACS1B*, *LeACS2*, *LeACS3*, *LeACS4*, *LeACS5*, *LeACS6*, *LeACS7*, *LeACS8*) and *ETR* (*LeETR1*, *LeETR2*, *LeETR3*, *LeETR4*, *LeETR5*, *LeETR6*) were first obtained from the NCBI database ([47], accessed on 21 January 2013) before being used to identify any related capsicum accessions using the BLASTn search in the NCBI database as well as a capsicum EST database ([48], accessed on 21 January 2013) as per Aizat et al. [16]. The accession numbers are listed in Additional file 1: Table S1, and the corresponding phylogenetic analysis is presented in Additional file 2: Figure S1. Capsicum isoforms were named according to the corresponding homologues in tomato for easier reference and comparison. For the *ACO* isoforms, only a partial sequence of capsicum *CaACO1* from Garcia-Pineda and Lozoya-Gloria [32] and a full-length of *CaACO4* from Aizat et al. [16] were available in the NCBI database whereas all six possible capsicum isoforms were identified using the capsicum EST database which encoded for full length proteins, except *CaACO5*. For the *ACS* isoforms, only *CaACS1* and *CaACS2* accessions were identified that encode full-length proteins and one EST transcript (partial) that closely matched *LeACS3*. For the capsicum *ETR* isoforms, no NCBI accession was found that closely related to any tomato *ETR* isoform. However using the capsicum EST database, one EST closely matched to *LeETR3* (named *CaETR3*) and two ESTs related to *LeETR4* and *LeETR5* (named *CaETR4* and *CaETR5*, respectively) were identified (all partial).

In order to further isolate any other possible *ACS* and *ETR* isoforms, degenerate primers were designed based on consensus sequences of highly conserved regions in *LeACS3*, *LeACS4*, *LeACS5*, *LeACS6*, *LeACS7* and *LeACS8* for *ACS* and subfamily I (*LeETR1*, *LeETR2*, *LeETR3*) as well as subfamily II (*LeETR4*, *LeETR5*, *LeETR6*) receptors for *ETR*. Available EST sequences from capsicum were also taken into consideration when designing these degenerate primers. However, no PCR product (using pooled cDNA from six ripening stages as templates) was detected for *ACS* degenerate primers but both sets of primers for *ETR*s produced a single band (Additional file 3: Figure S2) which after sequencing contained two different products. Phylogenetic analysis reveals that *ETR* subfamily I primers yielded products closely related to *LeETR2* and *LeETR3* while *ETR* subfamily II primers yielded products that matched *LeETR4* and *LeETR5* (Additional file 2: Figure S1). Three of these sequences matched to the three earlier EST sequences (*CaETR3*, *CaETR4* and *CaETR5*) but one sequence which is related to *LeETR2* (hence named *CaETR2*) did not match to any annotations in any databases searched.

Primers specific for each capsicum isoform were designed and run in end-point PCR. All primer pairs resulted in the amplification of a single PCR product using the pooled cDNA as templates (Additional file 3: Figure S2) and were specific to each isoform based on sequencing and a single qPCR melt curve (data not shown). However, *CaACS3* was not expressed as its primer was able to amplify genomic DNA but not its cDNA transcript (Additional file 3: Figure S2). Furthermore, *ACS4* primers from Osorio et al. [14] were not able to amplify any products even from the genomic PCR (Additional file 3: Figure S2), with no other information on *CaACS4* available in the NCBI and EST databases. *CaGAPdH* primers were obtained from Ogasawara et al. [49] and used in Aizat et al. [16] as well.

### qPCR analysis

qPCR was performed as outlined by Schaarschmidt et al. [50]. Briefly, three biological replicates for each ripening stage and for different treatments at 0 DAT were analysed. A 1:10 dilution with double-sterilised nanopure water was made for all cDNA stocks and run in a qPCR instrument (ViiA™ 7, Life Technologies, USA). In each 10 μL qPCR reaction (three technical replicates for each sample), the diluted cDNA template (1.5 ng reverse transcribed total RNA) and primers (5 pmol of each forward and reverse) were mixed with SYBR® Green reagent (iQ™ supermix, BioRad, USA). The qPCR running conditions were 95°C for 15 s, 40 cycles of 95°C for 15 s and 55°C for 30 s, followed by one step of 95°C for 15 s, 60°C for 1 min and 95°C for 15 s to generate the melt curve. A positive control (using 1:1000 dilution of purified plasmid transformed with *CaGAPdH* PCR product as the template and corresponding primers) and negative controls of no template were run in all qPCR plates. Ct values for each reaction were evaluated using in-built ViiA™ 7 version 1.2 software (Applied Biosystems) and imported into the Microsoft Excel program. The relative gene expression analysis was done according to the standard comparative Ct method (2^-∆∆Ct^) by correcting the Ct values of each gene to the positive control, before normalisation of the gene of interest to the *CaGAPdH* endogenous control. Furthermore, samples which did not possess any significant melt curve across all three technical replicates were considered to not be expressing that particular isoform and a value of 0 relative expression was set.

### Enzymatic assays and colour (ASTA) measurement

The ACS activity assay was adapted from Kato et al. [51] with some modifications. Briefly, ground tissues [0.2 g FW] were homogenised with 1.8 mL ice-cold EPPS buffer A (0.1 M EPPS-KOH pH 8.5, 10 mM 2-mercaptoethanol, 10 μM pyridoxal phosphate). Samples were centrifuged at 13 000 rpm at 4°C for 20 min. A clear sample (0.5 mL) was added with an ice-cold EPPS buffer B (0.5 mL of 0.1 M EPPS-KOH pH 8.5, 0.2 mM SAM) in a test tube (12 × 75 mm) fitted with a rubber stopper (9.5 mm suba-seal). All reaction mixtures were incubated for 30 min at 30°C and the ACS activity was then measured as per Kato et al. [51]. Another reaction containing a spike solution (0.5 mL EPPS buffer B with 0.01 μM ACC) and 0.5 mL sample (extracted with the EPPS buffer A above) was also prepared similarly to calculate the efficiency of the assay as per Bulens et al. [52]. The ACC content was measured as per Tan et al. [20]. For both ACS activity and ACC content assays, ground tissues of one biological replicate of commercial ripe banana (Stage 5 according to CSIRO [53]) and red tomato were also run similarly as positive controls. The ACO activity assay was run as detailed in Aizat et al. [16].

Extractable colour was measured according to the standard method of the American Spice Trade Association, ASTA [54]. Ground tissues (1.5 g) were dried at 40°C for 24 h. Dried materials were weighed to approximately 43 mg, incubated in flasks containing 50 mL absolute acetone and shaken in the dark for 18 h. The absorbance of the extracted colour was determined at a wavelength of 460 nm using a UV/VIS Spectrophotometer SP 8001 (AdeLab Scientific, Thebarton, Australia) and the ASTA units were calculated according to the standard formula [54].

### Statistical analysis

All statistics were performed using Genstat 14 (Hemel Hempstead, UK). Least Significant Difference (LSD) at *P* < 0.05 in the Analysis of Variance (ANOVA) was used to determine significantly different means, unless otherwise stated. The statistical significance for the different treatments at each of the sampling times was determined using Duncan’s Multiple Range Test at *P* < 0.05 (Additional file 4: Table S2).

## Abbreviations

ACC: 1-aminocyclopropane 1-carboxylate; ACO: ACC oxidase; ACS: ACC synthase; ETR: Ethylene receptor; G: Green; B: Breaker; BR1: Breaker red 1; BR2: Breaker red 2; LR: Light red; DR: Deep red.

## Competing interests

The authors declare that they have no competing interests.

## Authors’ contributions

WMA performed the experiments. WMA, JAA, JCRS and AJA participated in the design and interpretation of the study, all authors read and approved the final manuscript.

## Supplementary Material

Additional file 1: Table S1 List of primers used in PCR and qPCR. Click here for file

Additional file 2: Figure S1 **The phylogenetic analysis of ACO (A), ACS (B) and *ETR *(C and D) isoforms.** The tree was built based on full-length protein sequences of ACO isoforms (except CaACO5 which is partial), and the full length of ACS isoforms. The trees for *ETR*s were built based on mRNA sequences of tomato and the partial sequence of capsicum *ETR*s subfamily I (*CaETR2* and *CaETR3*, C) and subfamily II (*CaETR4* and *CaETR5*, D) obtained from an end point RT-PCR using degenerate primers for respective subfamilies (refer to Methods). Genbank accession numbers for the tomato are: LeACO1, P05116.2; LeACO2, CAA68538.1; LeACO3, CAA90904.1; LeACO4, NP_001233867.1; LeACO5, NP_001234037.1; LeACO6, ABP68407.1; LeACS1A, NP_001233922.1; LeACS1B, AAB17279.1; LeACS2, NP_001234178.1; LeACS3, NP_001234026.1; LeACS4, NP_001233875.1; LeACS5, NP_001234156.1; LeACS6, BAA34923.1; LeACS7, NP_001234346.1; LeACS8, NP_001234160.1; *LeETR1*, NM_001247220.1; *LeETR2*, NM_001247224.1; *LeETR3*, NM_001246965.1; *LeETR4*, NM_001247276.1; *LeETR5*, NM_001247283.1 and *LeETR6*, NM_001247221.1. For capsicum, the available Genbank accession numbers are: CaACO4, AGG20315; CaACS1, BAG30909.1; and CaACS2, BAG30910.1. All other capsicum isoforms (from contigs of the EST database) are listed in Additional file 1: Table S1 translated *in silico* as per [16]. Le, tomato; Ca, Capsicum. Click here for file

Additional file 3: Figure S2 End-point PCR for isolating capsicum *ACO*, *ACS* and *ETR* isoforms. Degenerate primers of *ETR* subfamily I (*ETRdeg1-*3) and subfamily II (*ETRdeg4*-6) as well as *ACS* isoforms (*ACSdeg*) were run in the end-point PCR (using cDNA pooled from six stages of ripening) to isolate other possible isoforms due to the lack of information in the databases (A). The primers for six *CaACO* isoforms (B), four *CaACS* isoforms (C) and four *CaETR* isoforms (D) were run in the end-point PCR using the pooled cDNA template. All bands are less than 0.26 kb using primers listed in Additional file 1: Table S1. The differences between band intensity (especially *CaACO1* and *CaETR4*) could be due to loading. The absence of *CaACS3* and *CaACS4* in the cDNA mix was also confirmed in independent RT-PCR and qPCR experiments (data not shown). (E) Genomic PCR using DNA template (annealing temperature 60°C) was also performed but only the *CaACS3* (and positive control *CaGAPdH* plus introns) produced specific products and not *CaACS4*. Experiments were repeated using two other annealing temperatures (55°C and 65°C), again without any amplification of *CaACS4* (data not shown). *CaACS4* primers were obtained from a previous qPCR study in capsicum and tomato [14] and no other information regarding its sequence was available in either the NCBI or capsicum EST databases. Click here for file

Additional file 4: Table S2 Significant levels of colour (Figure 2B), ACO activity (Figure 3A), ACS activity (Figure 3B) and ACC content (Figure 3C) were determined using Duncan’s Multiple Range Test (*P* < 0.05). This analysis compares all data for control (C), ethylene (E) and 1-MCP (M) treated fruit at different days after treatment (DAT) within the respective ripening stage. Click here for file
