# Supplementary material for: Characterisation of ethylene pathway components in non-climacteric capsicum
Source: BMC Plant Biol. 2013 Nov 28;13:191. doi: 10.1186/1471-2229-13-191 (PMC4219378; doi:10.1186/1471-2229-13-191)
Supplement: Additional file 1: Table S1 — List of primers used in PCR and qPCR. [file 1471-2229-13-191-S1.pdf]

Additional file 1: Table S1. List of primers used in PCR and qPCR.

**Gene/ isoform names**	**NCBI accession numbers for tomato (*Le*)**	**Contig accession numbers for capsicum (*Ca*)[g]**	**NCBI accession numbers for capsicum (*Ca*)**	**Forward primer (5'-3')**	**Reverse primer (5'-3')**	**Product size**
GAPdH[a]	-	-	AJ246011.1	GAAGAATTGGTCGATTGGTG	CCCCGTTGACTCTACGACAT	272
ACO1	NM_001247095.1	TC13435	GD053075.1 & GD054188.1	GCAAGTGCTTAAATTACAAGTGTG	TTGAGATGCAACCGTTACTCC	195
ACO2	XM_004251479.1	TC14180	GD052945.1, GD054373.1 & CA520300.1	GTTGAGAAAGAGGCAAAGGAT	AAATAATCTTGAGTTTGAATGTACG	188
ACO3	NM_001246999.1	TC12536	GD095125.1 & BM063409.1	ATCCAGCACCAGCTTTGATC	TGAATTGGAAATTAAGCACTTG	193
ACO4	AB013101.1	-	JX515597	GCAATGAAGGCTATGGAGACTA	TGACGCATCAAGAAACACAAG	217
ACO5[b]	AJ715790.1	TC15257	GD115323.1& GD117950.1	GGACATTCCACCTTCCAAGA	AGAGCTTTGGTGCTGGAGAA	188
ACO6	EF501822.1	TC24188	GD089267.1, GD124776.1, GD101463.1 & GD099075.1	GGAGCCAAGGTTTGAAGCA	CATCACACTACCAGACAACAGTACA	259
ACS1[c]	NM_001246993.1 (A) and U72390.1 (B)	-	AB434926.1	AAGTTGTCGATGAAATGATTGG	CTAGCGATGTCCACTACTGTATCA	136
ACS2[c]	NM_001247249.1	-	AB434927.1	GTAGAAGACAACGAAATGAGTCAT	CGCAACTAACGACAGCATC	133
ACS3[b]	NM_001247097.1	KS23061E03	GD116714.1	CTTGAACTCCTCCTTACATTCG	AACTCGTTCCCAAACATCTGT	156
ACS4[d]	NM_001246946.1	-	-	CCATCTTGTTTGCGACGAAATA	CGATGCTAACGAATTTTGGAGAA	-
ACSdeg[f]	accordingly	-	-	ATGGGTNTNGCNGAAAATCA	AANGCATCNCCNGGNTNAG	287
ETR2[e]	NM_001247224.1	-	-	GGAACCAATGAGGGCTCG	CAGCCAGGAAATCATTACGC	107
ETR3[b]	NM_001246965.1	KS16036C11	GD072810.1	AGAGTCCATGCGAGCCCA	CTTCTTGTCGAGCTACATCCAA	68
ETR4[b]	NM_001247276.1	KS20049F06 & TC15606	GD097854.1 & GD101436.1	TTGGGATGCAGCGTGTCT	AAGGACGATTTGGAATGAGG	87
ETR5[b]	NM_001247283.1	TC12426	GD063530.1 & GD052924.1	CTTCATGTACTCGTTGCTGATG	CTAAAGCACTCAGGCATTGG	121
ETR1-3deg[f]	accordingly	-	-	AGATCAGGTNGCTGTNGCTC	GCATNGGNGTTCNCATTTCAT	179
ETR4-6deg[f]	accordingly	-	-	TGATGATGTNAATAGANNGGTAAC	TCNGGCANNTGAAGATCCAA	150

[a]*CaGAPdH* primers were obtained from other studies detailed in the manuscript.

[b]Contig sequences for *CaACO5*, *CaACS3*, *CaETR3*, *CaETR4* and *CaETR5* are partial and not encoded for full-length proteins.

[c]*CaACS1* and *CaACS2* sequences are from *Capsicum chinense* obtained through the NCBI database (similar EST sequences were also obtained from the capsicum EST database). For the tomato *ACS1*, there are two different isoforms exist named *LeACS1A* and *LeACS1B*. However, only one isoform is available for capsicum which is closely related to both tomato *ACS1* isoforms (Additional file 2: Figure S1). Hence, the capsicum *ACS1* is named *CaACS1*.

[d]Primers for *CaACS4* were obtained from previous qPCR study in capsicum and no other information regarding its sequences are available in both NCBI and EST databases (detailed in manuscript).

[e]*CaETR2* was extracted from the degenerate primers (ETR1-3deg).

[f]Degenerate primers for *ACS* (*ACSdeg*) were designed based on consensus sequence of *LeACS3*, *LeACS4*, *LeACS5*, *LeACS6*, *LeACS7* and *LeACS8* and the degenerate primers for *ETR* (*ETR1-3deg* and *ETR4-6deg*) were designed based on the consensus sequences of tomato Type I (*LeETR1*, *LeETR2*, *LeETR3*) and Type II (*LeETR4*, *LeETR5*, *LeETR6*), respectively. Any available capsicum EST sequences were also taken into consideration when designing these primers.

[g]Contig accession numbers were obtained from the EST database which are linked to the NCBI dbEST database (some of them have more than one dbEST accessions as listed in Genbank accession numbers column of the table).
