# Supplementary material for: Characterisation of ethylene pathway components in non-climacteric capsicum
Source: BMC Plant Biol. 2013 Nov 28;13:191. doi: 10.1186/1471-2229-13-191 (PMC4219378; doi:10.1186/1471-2229-13-191)
Supplement: Additional file 2: Figure S1 — The phylogenetic analysis of ACO (A), ACS (B) and ETR (C and D) isoforms. The tree was built based on full-length protein sequences of ACO isoforms (except CaACO5 which is partial), and the full length of ACS isoforms. The trees for ETRs were built based on mRNA sequences of tomato and the partial sequence of capsicum ETRs subfamily I (CaETR2 and CaETR3, C) and subfamily II (CaETR4 and CaETR5, D) obtained from an end point RT-PCR using degenerate primers for respective subfamilies (refer to Methods). Genbank accession numbers for the tomato are: LeACO1, P05116.2; LeACO2, CAA68538.1; LeACO3, CAA90904.1; LeACO4, NP_001233867.1; LeACO5, NP_001234037.1; LeACO6, ABP68407.1; LeACS1A, NP_001233922.1; LeACS1B, AAB17279.1; LeACS2, NP_001234178.1; LeACS3, NP_001234026.1; LeACS4, NP_001233875.1; LeACS5, NP_001234156.1; LeACS6, BAA34923.1; LeACS7, NP_001234346.1; LeACS8, NP_001234160.1; LeETR1, NM_001247220.1; LeETR2, NM_001247224.1; LeETR3, NM_001246965.1; LeETR4, NM_001247276.1; LeETR5, NM_001247283.1 and LeETR6, NM_001247221.1. For capsicum, the available Genbank accession numbers are: CaACO4, AGG20315; CaACS1, BAG30909.1; and CaACS2, BAG30910.1. All other capsicum isoforms (from contigs of the EST database) are listed in Additional file 1: Table S1 translated in silico as per [16]. Le, tomato; Ca, Capsicum. [file 1471-2229-13-191-S2.pdf]

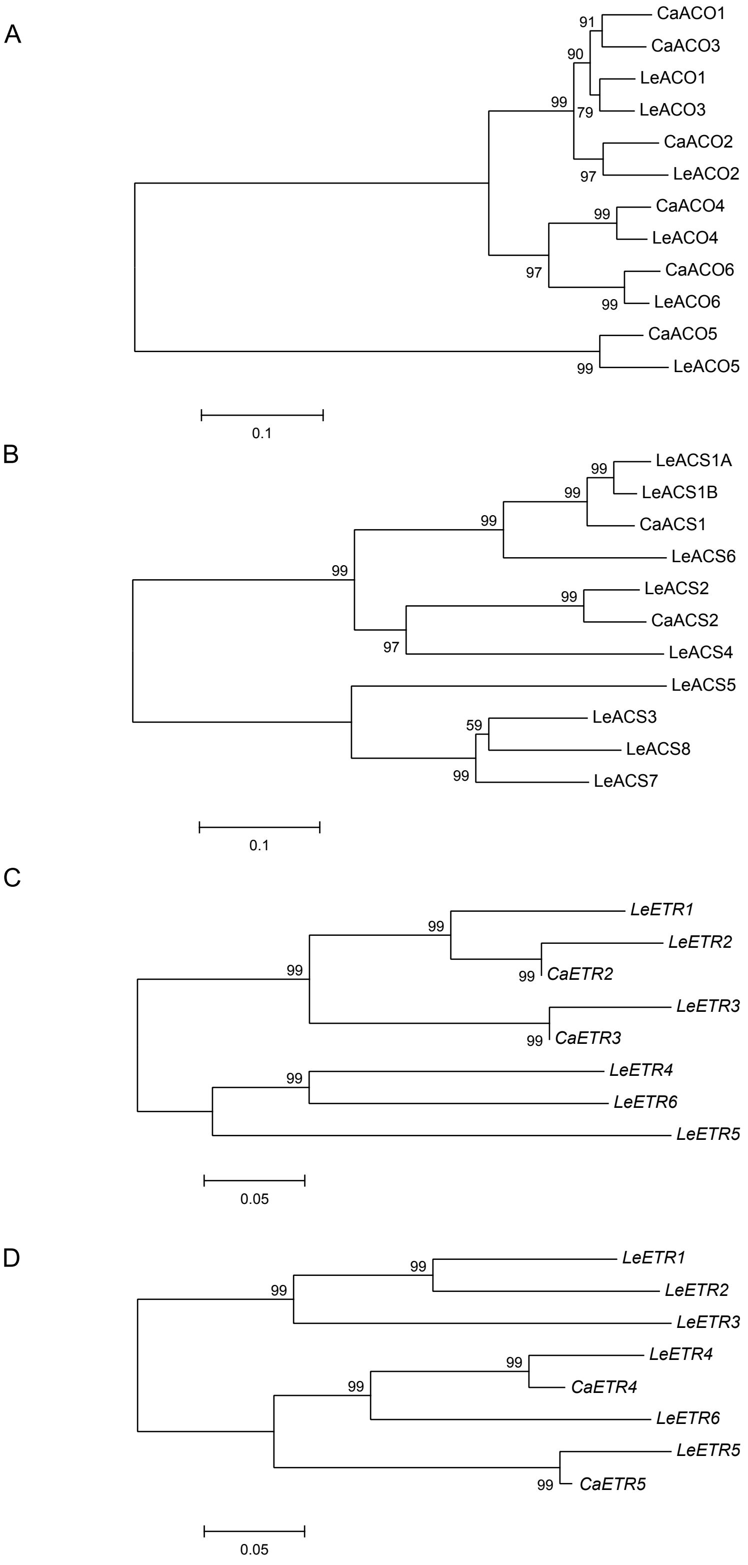

Additional file 2:Figure S1. The phylogenetic analysis of ACO (A), ACS (B) and *ETR* (C and D) isoforms. The tree was built based on full-length protein sequences of ACO isoforms (except CaACO5 which is partial), and the full length of ACS isoforms. The tree for *ETR*s was built based on mRNA sequences of tomato and the partial sequence of capsicum *ETR*s Type I (*CaETR2* and *CaETR3*, C) and Type II (*CaETR4* and *CaETR5*, D) obtained from an end point RT-PCR using degenerate primers for respective types (refer to Methods). Genbank accession numbers for the tomato are: LeACO1, P05116.2; LeACO2, CAA68538.1; LeACO3, CAA90904.1; LeACO4, NP_001233867.1; LeACO5, NP_001234037.1; LeACO6, ABP68407.1; LeACS1A, NP_001233922.1; LeACS1B, AAB17279.1; LeACS2, NP_001234178.1; LeACS3, NP_001234026.1; LeACS4, NP_001233875.1; LeACS5, NP_001234156.1; LeACS6, BAA34923.1; LeACS7, NP_001234346.1; LeACS8, NP_001234160.1; *LeETR1*, NM_001247220.1; *LeETR2*, NM_001247224.1; *LeETR3*, NM_001246965.1; *LeETR4*, NM_001247276.1; *LeETR5*, NM_001247283.1 and *LeETR6*, NM_001247221.1. For capsicum, the available Genbank accession numbers are: CaACO4, AGG20315; CaACS1, BAG30909.1 and CaACS2, BAG30910.1. All other capsicum isoforms (from contigs of the EST database) are listed in Additional file 1: Table S1 translated *in silico* as per [16]. Le, tomato; Ca, Capsicum.
