# Supplementary material for: Characterisation of ethylene pathway components in non-climacteric capsicum
Source: BMC Plant Biol. 2013 Nov 28;13:191. doi: 10.1186/1471-2229-13-191 (PMC4219378; doi:10.1186/1471-2229-13-191)
Supplement: Additional file 3: Figure S2 — End-point PCR for isolating capsicum ACO, ACS and ETR isoforms. Degenerate primers of ETR subfamily I (ETRdeg1-3) and subfamily II (ETRdeg4-6) as well as ACS isoforms (ACSdeg) were run in the end-point PCR (using cDNA pooled from six stages of ripening) to isolate other possible isoforms due to the lack of information in the databases (A). The primers for six CaACO isoforms (B), four CaACS isoforms (C) and four CaETR isoforms (D) were run in the end-point PCR using the pooled cDNA template. All bands are less than 0.26 kb using primers listed in Additional file 1: Table S1. The differences between band intensity (especially CaACO1 and CaETR4) could be due to loading. The absence of CaACS3 and CaACS4 in the cDNA mix was also confirmed in independent RT-PCR and qPCR experiments (data not shown). (E) Genomic PCR using DNA template (annealing temperature 60°C) was also performed but only the CaACS3 (and positive control CaGAPdH plus introns) produced specific products and not CaACS4. Experiments were repeated using two other annealing temperatures (55°C and 65°C), again without any amplification of CaACS4 (data not shown). CaACS4 primers were obtained from a previous qPCR study in capsicum and tomato [14] and no other information regarding its sequence was available in either the NCBI or capsicum EST databases. [file 1471-2229-13-191-S3.pdf]

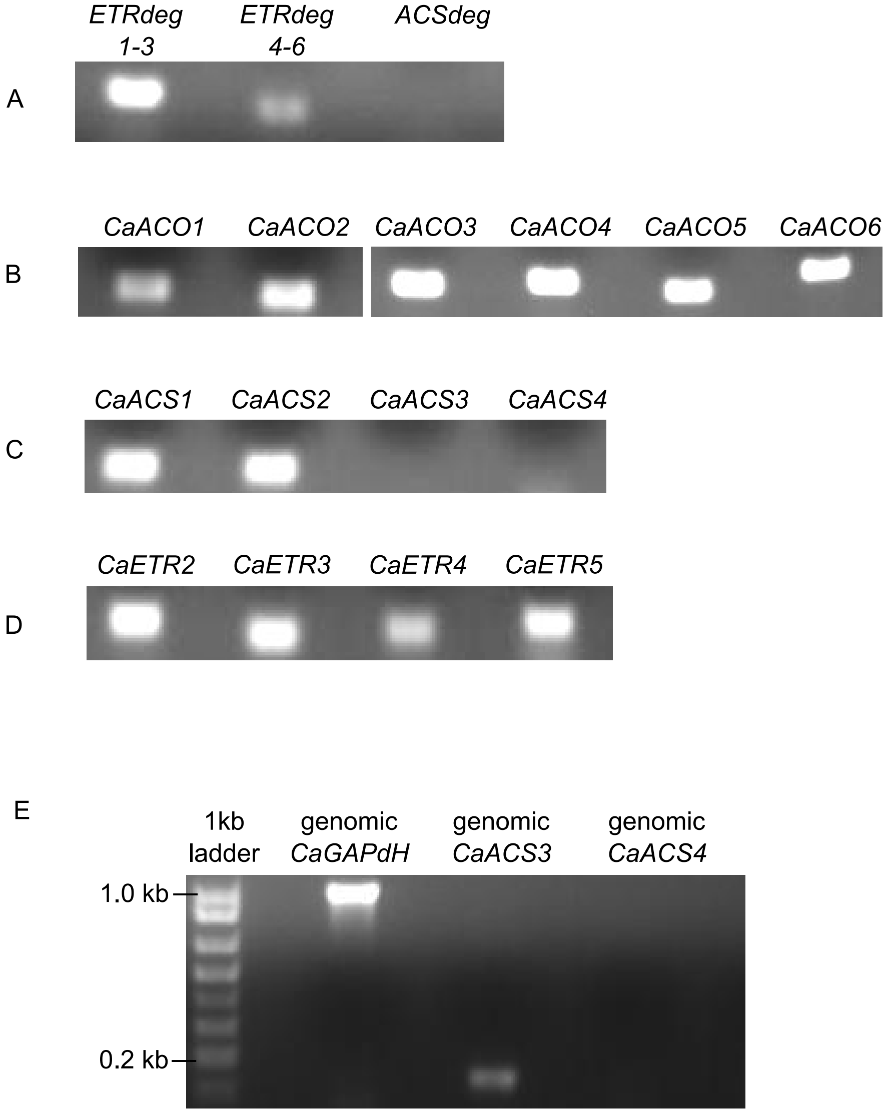

Additional file 3: Figure S2. End-point PCR for isolating capsicum *ACO*, *ACS* and *ETR* isoforms. Degenerate primers of *ETR* Type I (*ETRdeg1-3*) and Type II (*ETRdeg4-6*) as well as *ACS* isoforms (*ACSdeg*) were run in the end-point PCR (using cDNA pooled from six stages of ripening) to isolate other possible isoforms due to the lack of information in the databases (A). The primers for six *CaACO* isoforms (B), four *CaACS* isoforms (C) and four *CaETR* isoforms (D) were run in the end-point PCR using the pooled cDNA template. All bands are less than 0.26 kb using primers listed in Additional file 1: Table S1. The differences between band intensity (especially *CaACO1* and *CaETR4*) could be due to loading. The absence of *CaACS3* and *CaACS4* in the cDNA mix was also confirmed in another independent RT-PCR and qPCR experiments (data not shown). (E) Genomic PCR using DNA template (annealing temperature 60°C) was also performed but only the *CaACS3* (and positive control *CaGAPdH* plus introns) produced specific products and not *CaACS4*. Experiments were repeated in two other annealing temperatures (55°C and 65°C), again without any amplification of *CaACS4* (data not shown). *CaACS4* primers were obtained from a previous qPCR study in capsicum and tomato [14] and no other information regarding its sequence was available in either the NCBI or capsicum EST databases.
