# Supplementary material for: Characterisation of ethylene pathway components in non-climacteric capsicum
Source: BMC Plant Biol. 2013 Nov 28;13:191. doi: 10.1186/1471-2229-13-191 (PMC4219378; doi:10.1186/1471-2229-13-191)
Supplement: Additional file 4: Table S2 — Significant levels of colour (Figure 2B), ACO activity (Figure 3A), ACS activity (Figure 3B) and ACC content (Figure 3C) were determined using Duncan’s Multiple Range Test (P < 0.05). This analysis compares all data for control (C), ethylene (E) and 1-MCP (M) treated fruit at different days after treatment (DAT) within the respective ripening stage. [file 1471-2229-13-191-S4.pdf]

Additional file 4: Table S2. Significant levels of ASTA level (Figure 2B), ACO activity (Figure 3A), ACS activity (Figure 3B) and ACC content (Figure 3C) were determined using Duncan's Multiple Range Test (*P*<0.05). This analysis compares all data for control (C), ethylene (E) and 1-MCP (M) treated fruit at different days after treatment (DAT) within the respective ripening stage.

Stages	DAT	ASTA			ACO activity			ACS activity			ACC content		
		C	E	M	C	E	M	C	E	M	C	E	M
Green	0	a	a	a	bcde	ab	ef	a	a	a	ab	a	cde
	3	a	a	a	cdef	cdef	f	ab	a	a	ab	abc	abc
	12	a	a	a	bc	bc	bcd	a	a	ab	abcd	abcd	bcde
	20	a	ab	a	a	a	ab	c	bc	c	cde	bcde	abcde
	28	bc	c	c	cdef	def	f	cd	d	cd	de	f	ef
Breaker	0	ab	a	a	a	b	c	abcd	ab	ab	a	a	a
	3	cd	cde	abc	de	ef	cd	ab	a	abc	a	a	a
	12	ef	def	bcd	fg	fg	fg	abcd	abcd	abcd	ab	a	abc
	20	g	g	f	g	g	g	cd	d	abcd	abc	bcd	ab
	28	g	g	g	g	g	g	abcd	abcd	bcd	d	abc	cd
